# Upper Extremity Deep Vein Thromboses: The Bowler and the Barista

**DOI:** 10.1155/2016/9631432

**Published:** 2016-10-09

**Authors:** Seth Stake, Anne L. du Breuil, Jeremy Close

**Affiliations:** ^1^Sidney Kimmel Medical College, Thomas Jefferson University, Philadelphia, PA 19107, USA; ^2^Department of Family and Community Medicine, Sidney Kimmel Medical College, Thomas Jefferson University, 833 East Chestnut Street, Suite 301, Philadelphia, PA 19107, USA

## Abstract

Effort thrombosis of the upper extremity refers to a deep venous thrombosis of the upper extremity resulting from repetitive activity of the upper limb. Most cases of effort thrombosis occur in young elite athletes with strenuous upper extremity activity. This article reports two cases who both developed upper extremity deep vein thromboses, the first being a 67-year-old bowler and the second a 25-year-old barista, and illustrates that effort thrombosis should be included in the differential diagnosis in any patient with symptoms concerning DVT associated with repetitive activity. A literature review explores the recommended therapies for upper extremity deep vein thromboses.

## 1. Introduction

Effort thrombosis of the upper extremity refers to a deep venous thrombosis of the upper extremity resulting from repetitive activity of the upper limb [1]. While most cases of effort thrombosis occur in young elite athletes with strenuous upper extremity activity, any repetitive activity capable of damaging vascular endothelium can cause it. This article reports two cases: the first is of a 67-year-old patient who developed upper extremity deep venous thrombosis (UEDVT) after bowling and the second case is of a 25-year-old barista, whose job involved lifting 30-pound bags of coffee over her head, who developed thrombosis of the right central subclavian and axillary vein. These cases illustrate that effort thrombosis should be included in the differential diagnosis in any patient with symptoms concerning deep vein thrombosis (DVT) associated with repetitive activity.

## 2. Case #1

**Figure 1:**
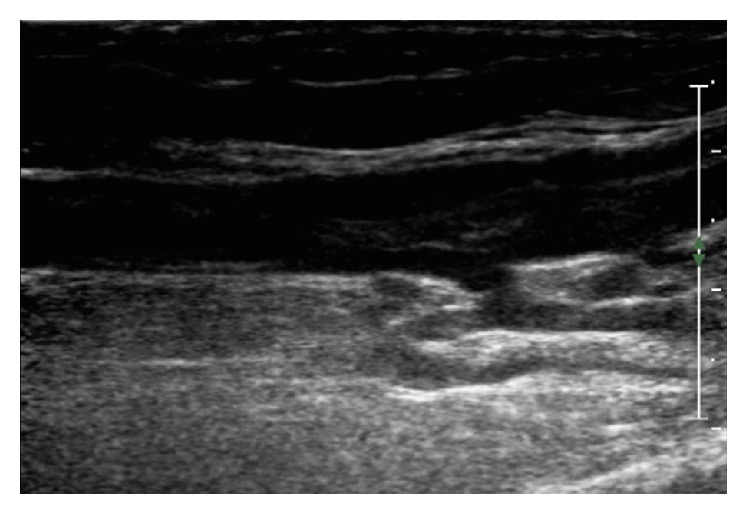
Right upper extremity ultrasound of a 67-year-old man revealing acute venous thrombosis in the brachial vein and peripheral axillary vein with acute superficial thrombosis in the right basilica vein.

A 67-year-old man experienced swelling in his proximal right upper extremity the morning after a prolonged game of bowling. He was an avid bowler and part of a competitive league. Over the next three days, the swelling became more prominent and was beginning to cause moderate-to-severe pain and so he was seen in our office. He denied any shortness of breath, chest pain, or history of trauma to his right arm. His past medical history was significant for a heart transplant 11 years earlier due to severe dilated cardiomyopathy that was complicated by thrombosis in his right internal jugular vein and right common femoral vein. He had not been diagnosed with a DVT since that hospital admission and denied any personal or family history of hematologic disease. On physical examination, he had an ill-defined 3 cm mobile, tender mass located deep to the subcutaneous space near the medial aspect of his right bicep. Ultrasound of the right upper extremity revealed acute deep venous thrombosis in the right brachial vein and peripheral axillary vein and acute superficial thrombosis in the right basilic vein (Figure 1).

The patient, who is on tacrolimus secondary to his heart transplant, was treated urgently with enoxaparin and then bridged to warfarin as an outpatient. After completing five weeks of outpatient anticoagulation therapy with warfarin, the patient denied any swelling or discomfort of the right upper extremity. Ultrasound evaluation showed that the thrombi had completely resolved. A sequential musculoskeletal ultrasound did not show any occlusion of blood vessels due to muscle hypertrophy. He was continued on anticoagulation and was approved to continue bowling as tolerated.

## 3. Case #2

**Figure 2:**
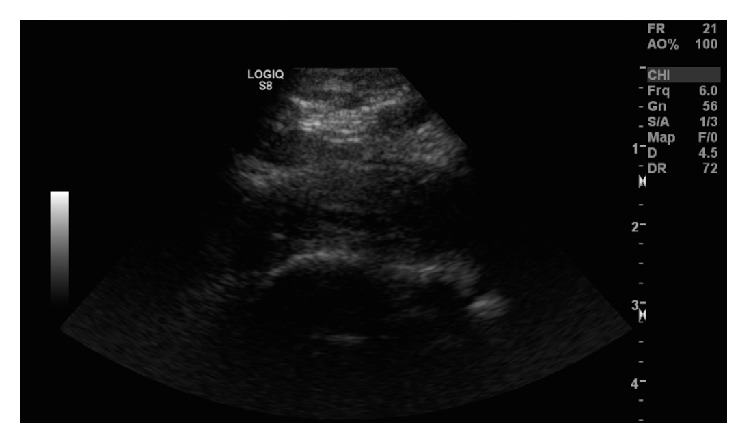
Right upper extremity ultrasound of a 25-year-old barista revealing acute thrombosis of the right central subclavian and axillary veins with compression by the first rib.

A 25-year-old otherwise healthy woman presented with a four-day history of right arm pain, swelling, heaviness, and redness. She was seen in an urgent care center and then sent to the emergency room for an ultrasound which showed acute thrombosis of the right central subclavian and axillary vein with compression by the first rib (Figure 2). She was discharged by the emergency room on rivaroxaban and advised to follow up at our office. On reviewing her history prior to her visit her physicians were concerned for possible thoracic outlet syndrome and obtained an emergency appointment with the vascular center. The patient had no prior history of DVT or pulmonary embolism (PE). Her job is that of a coffee roaster, and she lifts 30 pounds of coffee over her head all day. She denied any injuries, chest pain, shortness of breath, or lower leg pain or swelling. She also denied any history of any upper extremity catheters.

**Figure 3:**
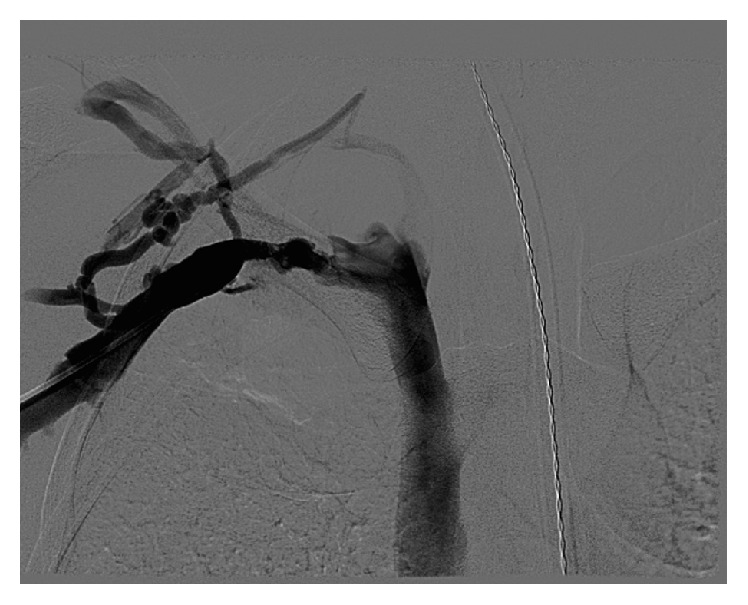
Preoperative angiogram of the right upper extremity revealing attenuation of contrast flow at the level of the subclavian vein secondary to thrombosis.

**Figure 4:**
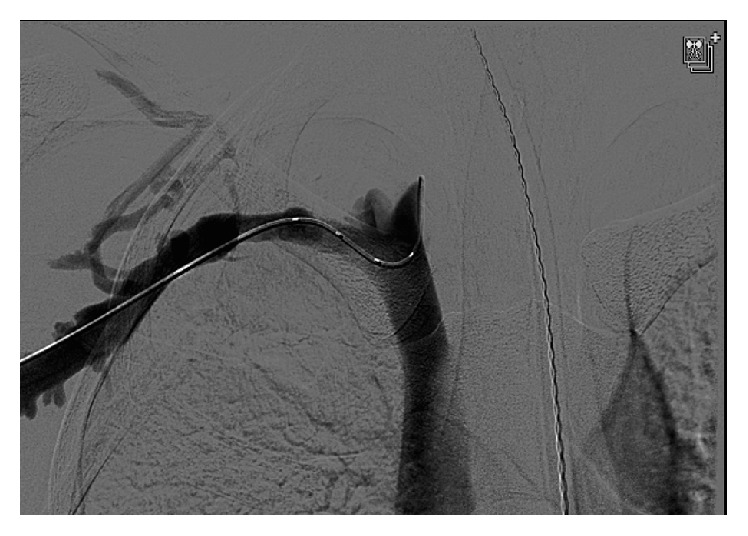
Angiogram revealing contrast flow through the subclavian vein status after catheter-directed thrombolysis and balloon angioplasty of the subclavian and axillary veins.

Patient was admitted and placed on a heparin drip. The next day she underwent catheter-directed thrombolysis, 24 hours of lysis, and then balloon angioplasty of the right subclavian and axillary veins. After thrombolysis a venogram was performed with the arm abducted, and it showed that the contrast was still not flowing through the subclavian vein (Figures 3 and 4). Then another venogram was done with the arm adducted and it showed that the junction between the first rib and the clavicle was opened and there was flow through the subclavian vein. At that point there was not an obvious filling defect in the subclavian and axillary vein. Two days later she underwent resection of her first rib. After stabilization the patient was discharged on rivaroxaban 15 mg bid for 21 days and then switched to rivaroxaban 20 mg daily. She was seen three weeks later and denied right upper extremity pain, paresthesias, or numbness. She denied any bleeding or bruising, shortness of breath, fever, or chills. She was advised to follow up at 3 months for a follow-up venogram and anticoagulation management.

## 4. Discussion

Compared to lower extremity DVT, upper extremity DVT (UEDVT) is quite rare, accounting for less than 3% of all venous thromboses [2, 3]. UEDVT is typically classified as being either primary (spontaneous) or secondary in origin. Primary UEDVT is a much rarer disorder (2 per 100,000 persons per year) that refers to either effort thrombosis or idiopathic UEDVT [2, 4, 5]. Secondary UEDVT occurs in patients with known risk factors such as central venous catheters, pacemakers, cancer, or other thrombophilic states. Pulmonary embolism is present in up to one-third of patients with UEDVT and other associated complications such as loss of vascular access, superior vena cava syndrome, and persistent pain and swelling can be devastating [4, 6].

Effort thrombosis is the phenomenon of a deep venous thrombosis in the upper extremity secondary to repetitive upper limb activity. The condition, first described in 1949, classically refers to axillary-subclavian vein thrombosis and is also known as Paget-Schroetter syndrome (PSS). Paget-Schroetter syndrome is a form of thoracic outlet obstruction, which refers to the compression of the neurovascular bundle (brachial plexus, subclavian artery, and subclavian vein) as it exits the thoracic inlet. Effort thrombosis usually follows high-intensity sporting activities such as baseball, wrestling, and swimming. This syndrome occurs in young athletes with hypertrophied muscles or in patients with anatomic abnormalities that constrict the thoracic outlet (cervical rib, hypertrophy of anterior and medial scalene muscles, and abnormal insertion of the costoclavicular ligament) [7]. This leads to compression of the vein, damage to the endothelium, and activation of the coagulation cascade. The repeated trauma to the vessel can result in fibrous tissue formation that persistently compresses the vein and can lead to a cycle of endothelial trauma, thrombosis, and recanalization. Progressive fibrosis of the vascular endothelium may result in extensive collateral formation [3].

Clinically, PSS typically involves the dominant arm. It usually presents in the asymptomatic healthy athlete. When symptomatic, it most frequently presents with swelling and pain [6]. Patients frequently are unable to pinpoint a discrete precipitating event but usually have some form of repetitive upper limb activity. Diagnostic ultrasound is the initial imaging test of choice for diagnosing UEDVT. It is inexpensive, noninvasive, reproducible modality, but it may fail to detect central thrombus if located directly below the clavicle due to acoustic shadowing [6, 8]. Conservative treatment of UEDVT with anticoagulation alone can lead to pulmonary embolism in 6–15% and has a high incidence of recurrent thrombosis and residual venous obstruction in up to 75% of the cases [7].

More aggressive treatment modalities, such as systemic fibrinolysis, are superior to anticoagulation in terms of achieving vein patency but are associated with higher risks of catastrophic bleeding [9]. However, catheter-directed thrombolysis has been reported to be successful in between 62% and 84% of cases provided the symptoms have persisted for less than 10–14 days [10]. Other modes of therapy have been directed at thoracic outlet decompression with resection of the first rib and division of the scalene muscles and the costoclavicular ligament [7, 8, 10, 11]. While some investigators see this treatment as first line, others only recommend it in resistant cases following failed treatment with anticoagulation or fibrinolysis [10]. Research is currently aiming to delineate the factors predicting the need for more aggressive treatments, such as thoracic outlet decompression and catheter-directed thrombolysis [10]. All patients are anticoagulated for 3–6 months after canalization and decompression.

Effort thrombosis has been studied for many years in patients with high-intensity upper arm movement and support that the recognition and urgent treatment of effort thrombosis are important to return these athletes to the field at an equal level of play. In a study done at Washington University four cases of effort thrombosis in major league baseball players diagnosed using contrast venography were all treated with catheter-directed thrombolysis, first-rib resection, and systemic anticoagulation. All four players returned to play at their previous level of competition [12]. Other studies support the recognition of effort thrombosis to prevent catastrophic injury such as the case of a 25-year-old major league pitcher who presented with dizziness and shortness of breath without upper extremity symptoms. He was diagnosed with effort thrombosis and secondary pulmonary embolus and treated successfully with mechanical thrombectomy and catheter-directed venolysis—a lifesaving intervention [1].

These cases represent classic clinical presentations of UEDVT (pain and swelling of the upper extremity). However, while the barista presents with classic PSS, the bowler's three UEDVT start in the middle of his biceps. While the patient had undergone repetitive movements of the upper extremity a few days before, bowling is not typically thought of as being highly intense. A literature review reveals other patients diagnosed with effort thrombosis following mild activity. UEDVT has been diagnosed in patients following games of pool [13], stretching [14], and “Shake Weight” exercises [15]. The case of the pool player involved a 22-year-old pool player who developed UEDVT after a prolonged game of pool. This study suggested that any activity that extends and internally rotates the shoulder can stretch and possibly compress the subclavian vein against the first rib [13].

The bowler's case of effort thrombosis is particularly unusual not only due to mechanism but also due to location. Classically, the damage to the subclavian and axillary veins in PSS is located at the junction of the first rib, clavicle, and the anterior and middle scalene muscles. In the bowler, the axillary vein thrombus was located distal to this point, near the medial border of the biceps muscle. The bowler additionally had a DVT in the brachial vein and a superficial thrombosis in the axillary vein. While the axillary and subclavian veins are classically associated with effort thrombosis, brachial vein thrombosis is not typical [16]. UEDVT involving thrombi in all three of these veins is exceedingly rare. As part of his hypercoagulability workup, our patient needs monitoring for occult malignancy and other secondary causes of UEDVT. Up to one-fourth of patients with idiopathic UEDVT were later diagnosed with malignancy [17], and up to one-third of patients with UEDVT develop PE [18]. As his thrombosis was not located in the thoracic outlet surgical decompression was not an option for him. Therefore, it will be crucial to educate our patient not to hesitate to follow up with any recurrent swelling of the upper extremity or symptoms of pulmonary embolus.

In contrast, the barista is a case of classic PSS caused by thoracic outlet syndrome. As seen on her intraoperative venogram her vein was occluded when her arm was abducted even though the vein was clearly open when her arm was adducted. This case shows the urgent need to refer such patients to vascular surgery for thrombolysis and then resection of the first rib.

In conclusion, these cases illustrate two cases of UEDVT presenting in two patients with histories of repetitive use of their arms, one as a bowler and the other as a barista. Clinical awareness of UEDVT in a patient with recent repetitive upper extremity movement is vital to avoid potentially disastrous complications. In the case of the bowler, treatment with anticoagulation and strict follow-up for the development of hypercoagulable disorders or malignancy have allowed the patient to return to the bowling alley. In the second case urgent awareness of the need to obtain surgical intervention was needed. Even once the thrombosis in her subclavian vein had been removed, she still did not have circulation in her subclavian vein when her arm was abducted. That a surgical intervention almost did not occur was seen when the patient was sent home from the emergency ward on anticoagulation alone. Fortunately, she was seen and referred the next day and operated on expeditiously.
